# An Atypical Riboflavin Pathway Is Essential for *Brucella abortus* Virulence

**DOI:** 10.1371/journal.pone.0009435

**Published:** 2010-02-25

**Authors:** Hernán Ruy Bonomi, María Inés Marchesini, Sebastián Klinke, Juan E. Ugalde, Vanesa Zylberman, Rodolfo A. Ugalde, Diego J. Comerci, Fernando Alberto Goldbaum

**Affiliations:** 1 Fundación Instituto Leloir-CONICET, Buenos Aires, Argentina; 2 Instituto de Investigaciones Biotecnológicas-CONICET, Universidad Nacional de General San Martín, San Martín, Buenos Aires, Argentina; National Institutes of Health, United States of America

## Abstract

Brucellosis is a worldwide zoonosis that affects livestock and humans and is caused by closely related *Brucella spp.*, which are adapted to intracellular life within cells of a large variety of mammals. *Brucella* can be considered a furtive pathogen that infects professional and non-professional phagocytes. In these cells *Brucella* survives in a replicative niche, which is characterized for having a very low oxygen tension and being deprived from nutrients such as amino acids and vitamins. Among these vitamins, we have focused on riboflavin (vitamin B2). Flavin metabolism has been barely implicated in bacterial virulence. We have recently described that *Brucella* and other Rhizobiales bear an atypical riboflavin metabolic pathway. In the present work we analyze the role of the flavin metabolism on *Brucella* virulence. Mutants on the two lumazine synthases (LS) isoenzymes RibH1 and RibH2 and a double RibH mutant were generated. These mutants and different complemented strains were tested for viability and virulence in cells and in mice. In this fashion we have established that at least one LS must be present for *B. abortus* survival and that RibH2 and not RibH1 is essential for intracellular survival due to its LS activity *in vivo*. In summary, we show that riboflavin biosynthesis is essential for *Brucella* survival inside cells or in mice. These results highlight the potential use of flavin biosynthetic pathway enzymes as targets for the chemotherapy of brucellosis.

## Introduction

Brucellosis is a worldwide zoonosis that affects livestock and humans and is caused by closely related *Brucella spp.* which are adapted to intracellular life within cells of a large variety of mammals. The most pathogenic species for humans are *B. melitensis*, *B. suis* and *B. abortus* whose preferred host are goats, pigs and cattle respectively. Transmission to humans occurs mainly through the consumption of contaminated unpasteurized dairy products and through direct contact with infected animals. An estimated of 500,000 human infections per year still occur worldwide [1].

*Brucella* can be considered a furtive pathogen that infects professional and non-professional phagocytes. No classical virulence factors, such as exotoxins, cytolysins, capsules, fimbria, plasmids, lysogenic phages, resistant forms, antigenic variation, endotoxic lipopolysaccharide or apoptotic inducers have been described in *Brucella spp.* so far [2]. Progress in understanding the molecular pathogenesis of the disease, vaccine engineering and postgenomic approaches aimed at the discovery of new pathways used by this pathogen to modify the intracellular environment may lead to new preventive interventions.

Once internalized, *Brucella* resides within a membrane-bound compartment, the *Brucella*-containing vacuole (BCV), which sequentially interacts and fuses with early endosomes, lysosomes and further traffics to the endoplasmic reticulum (ER) to generate an ER-derived replicative niche [3], [4], [5]. After 12–24 h after cell entry *Brucella* extensively replicates without restricting basic cellular functions or inducing obvious damage to cells [6]. The replicative niche is characterized for having a very low oxygen tension and being deprived in nutrients such as amino acids and vitamins [2]. The true virulence factors of *Brucella* are a complex array of molecular determinants that confer the pathogen the metabolic ability to thrive in the harsh intracellular conditions allowing it to invade, resist intracellular killing, build the replicative niche and replicate.

Although riboflavin (vitamin B2) is the precursor of the essential flavin coenzymes FMN and FAD which participate in a myriad of biochemical reactions, flavin metabolism has been barely implicated in bacterial virulence. Flavoenzymes are involved in dozens of crucial cellular processes such as energy metabolism, RedOx reactions, detoxification and biosynthesis [7]. For example, FMN is required as an electron acceptor for dehydrogenases in the respiratory chain, and its deficiency induces increased levels of Cytochrome bd, a terminal oxidase known to work in microaerophilic conditions which is also present in *Brucella* [8]. Thus, it is likely that flavin metabolism would be related to pathogenesis since *Brucella* needs to survive to oxidative stress, microaerobic conditions and nutrient starvation and flavoenzymes or flavins themselves may be playing a key role in those processes.

The metabolic pathway of riboflavin in bacteria, plants and fungi has been described in some detail [9], [10]. Briefly, the biosynthesis of one riboflavin molecule requires one molecule of GTP and two molecules of ribulose-5-phosphate. The imidazole ring of GTP is hydrolytically opened, yielding a 4,5-diaminopyrimidine that is converted to 5-amino-6-ribitylaminouracil by a sequence of deamination, side chain reduction, and dephosphorylation. Condensation of 5-amino-6-ribitylaminouracil with 3,4-dihydroxy-2-butanone-4-phosphate obtained from ribulose-5-phosphate affords 6,7-dimethyl-8-ribityllumazine (lumazine). Dismutation of the lumazine derivative yields riboflavin and 5-amino-6-ribitylaminouracil, which is recycled in the biosynthetic pathway (see Figure S1). Animals incorporate riboflavin as a micronutrient from their diet or throughout saprophyte bacteria to synthesize riboflavin-derived cofactors flavin mononucleotide (FMN) and flavin adenine dinucleotide (FAD).

**Figure 1:**
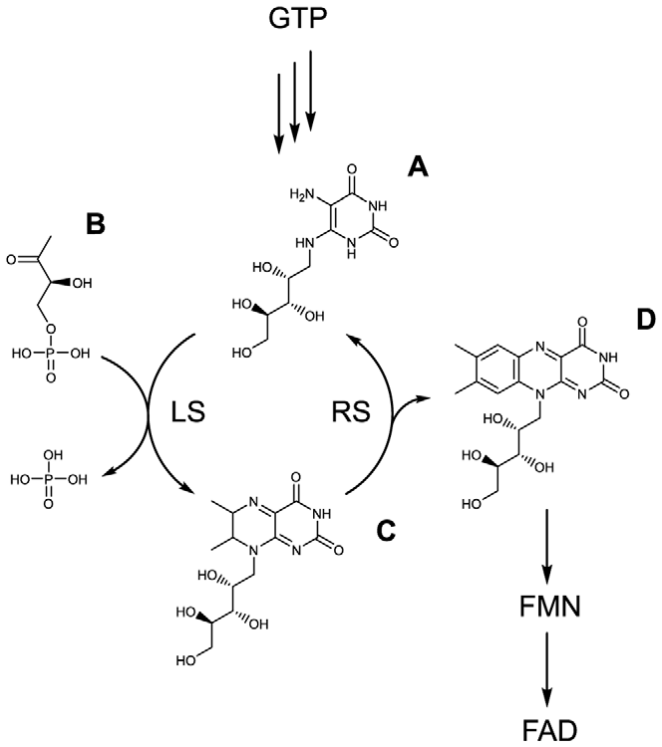
Bacterial, plant and fungal simplified riboflavin biosynthetic pathway. 5-amino-6-ribitylaminouracil (**A**) and 3,4-dihydroxy-2-butanone-4-phosphate (**B**) are condensed to give 6,7-dimethyl-8-ribityllumazine (lumazine) (**C**) by lumazine synthase **(LS)** during the penultimate step of the riboflavin biosynthesis. Riboflavin (**D**) is finally synthesized by riboflavin synthase **(RS)** which condensates two molecules of lumazine to yield one molecule of riboflavin and recycle one molecule of 5-amino-6-ribitylaminouracil into the pathway. *B. abortus* has two LSs, RibH1 and RibH2 which are Type-I and Type-II LSs respectively.

We have recently described that *Brucella* and other Rhizobiales bear an atypical riboflavin metabolic pathway [10], [11]. The enzyme 6,7-dimethyl-8-ribityllumazine synthase (lumazine synthase, LS) catalyzes the penultimate step in the biosynthesis of riboflavin (see Figure 1). A phylogenetic analysis on eubacterial, fungal and plant LSs allowed us to classify them into two categories: Type-I LSs (pentameric or icosahedral) and Type-II LSs (decameric). *Brucella* codes both a Type-I and a Type-II LS called RibH1 and RibH2 respectively. Both enzymes show low catalytic activity *in vitro*, suggesting that natural selection operating over the riboflavin pathway favored the evolution of catalysts with low reaction rates. The rationale behind this is that the excess of flavins in the intracellular pool in *Brucella* could act as a negative factor when these bacteria are exposed to oxidative or nitrosative stress [12].

In the present work we analyze the role of flavin metabolism on *B. abortus* intracellular survival, focusing at its two LS isoenzymes. Mutants on the *ribH1*, *ribH2* genes and a double *ribH* mutant were generated. These mutants and different complemented strains were tested for viability on cells and mice. In this fashion we have established that at least one LS must be present for *B. abortus* viability and that RibH2 and not RibH1 is neccesary for *Brucella* intracellular replication due to its LS activity *in vivo*. In summary, we show that flavin biosynthesis is essential for *Brucella* survival inside cells and mice.

## Results

### *Brucella* Needs at Least One *ribH* Gene to Survive

**Figure 2:**
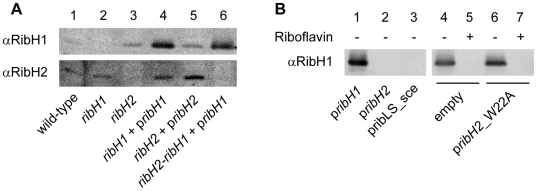
Western blot analysis of the mutants and complemented strains. (**A**) Wild-type *B. abortus* expresses both RibH1 and RibH2 in culture (lane 1). Mutant strains *ribH1* and *ribH2* present only one band corresponding to RibH2 and RibH1 respectively (lanes 2 and 3). Ectopic expression of RibH1 in *ribH1* and RibH2 in *ribH2* is confirmed in lanes 4 and 5 which in fact show higher amounts of these proteins than the wild-type strain. The chromosomal double *ribH* mutant only presents the RibH1 band corresponding to *ribH1* expressed from the rescue plasmid (lane 6). (**B**) Plasmid swap Western blots using anti-RibH1 antibodies. Lane 1 shows the positive control where p*ribH1*Km was replaced by p*ribH1*Amp. RibH1 could not be detected when p*ribH2* or pLS_sce were used to cure the p*ribH1*Km plasmid (lanes 2 and 3). Lanes 4 and 6 evidence that RibH1 expression was maintained when the pBB4 (empty) or p*ribH2*_W22A were used to replace p*ribH1*Km, but in the presence of riboflavin RibH1 was again not detected respectively (lanes 5 and 7).

In order to determine the role of the LS genes, simple and double mutants for the *ribH* genes were generated. The *ribH1* (BAB1_0791) mutant was made by clean non-polar deletion because this gene is believed to be located in an operon (rib operon). On the other hand, *ribH2* (BAB2_0545) is an isolated ORF, thus the *ribH2* mutant was generated by disrupting the *ribH2* gene with an antibiotic resistance cassette (see Material and Methods). Both simple mutants were obtained without any major difficulty and were confirmed by Western Blot analysis (Figure 2A, lanes 1–3). They are not auxotrophic for riboflavin and grow at wild-type rates in both rich (TSB) and minimal media (Gerhardt-Wilson) (data not shown). We then tried to obtain the double *ribH* mutant by disrupting *ribH2* in a *ribH1* mutant genetic context. All attempts were unsuccessful, suggesting that the double mutation might be lethal. To overcome this difficulty we changed our strategy and the *ribH1* mutant was complemented with a plasmid harboring *ribH1* under a constitutive promoter before *ribH2* mutagenesis (see materials and methods for details). In this way a chromosomal double *ribH* mutant (*ribH1-ribH2*) was obtained but at the expense of keeping a rescue copy of *ribH1* in an expression plasmid (Figure 2A, lanes 4 and 6). These results indicate that the double mutant *ribH1-ribH2* is not viable probably because it lacks a complete flavin biosynthesis pathway and that both genes encode LS activity but only one *ribH* gene would be sufficient for the bacteria to survive.

### RibH1 and RibH2 Have LS Enzymatic Activity *In Vivo*

**Figure 3:**
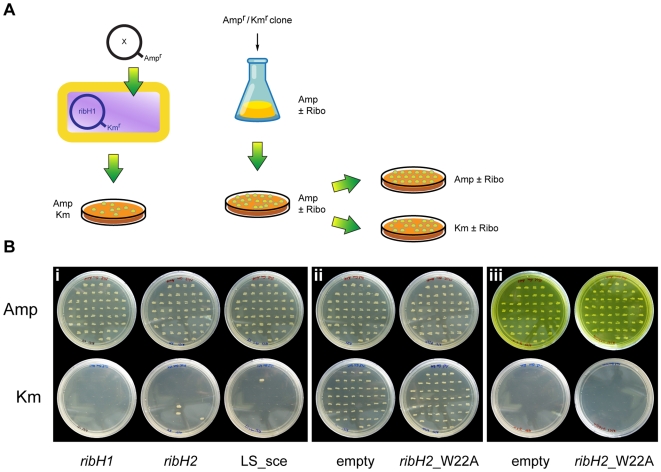
Both RibH1 and RibH2 have LS activity *in vivo*. (**A**) A schematic draft of the plasmid swap experiment is depicted. Different pBBR4 (Amp^r^) constructs were introduced by conjugation into the *ribH1-ribH2*+p*ribH1*Km strain (X: *ribH1*, *ribH2*, LS_sce, *ribH2*_W22A or an empty plasmid). Exconjugants were selected by Amp and Km resistance. Then, three independent clones from each conjugation were cultured separately in TSB with Amp for more than 15 generations. One hundred Amp^r^ clones were then replicated in Amp and Km plates and the percentage of Amp^r^/Km^s^ was determined. In the case when empty pBBR4 or p*ribH2*_W22A were used, riboflavin (Ribo) was added or not to the TSB and TSA to demonstrate that the presence of the final metabolite of the pathway could lead to the complete loss of LS activity in the cells. (**B**) The replica plates showed that more than 90% of the clones were Amp^r^/Km^s^ after the plasmid swap experiment when *ribH1*, *ribH2* or LS_sce inserts were tested (panel i). In the case of the *ribH2*_W22A insert or the empty plasmid (panel ii) less than 5% Amp^r^/Km^s^ clones were obtained. When riboflavin was added during the plasmid swap experiment more than 90% of the clones tested were Amp^r^/Km^s^ when p*ribH2*_W22A or pBBR4 (empty) were used to replace p*ribH1*Km (panel iii).

Given the above described mutagenesis studies and the fact that both RibH1 and RibH2 exhibit poor *in vitro* activity as LSs [11], [13], we decided to assess if they exhibit this enzymatic activity *in vivo*. To address this issue we designed a plasmid swap experiment assuming that the p*ribH1*Km plasmid that is harbored by the *ribH1-ribH2* strain is essential for the growth in TSB medium (poor in riboflavin) (Figure 2A). Thus we tested if the p*ribH1*Km plasmid could be cured in the presence of another plasmid from the same incompatibility group harboring different inserts but with an ampicillin resistance plasmid (pBBR4) (see Figure 3A for details). Theoretically, only plasmids that encode active LSs would be able to replace the original p*ribH1Km* plasmid after several generations. This is because there is a metabolic selective pressure for keeping LS enzymatic activity when bacteria are grown in riboflavin poor media. Figure 3Bi shows that RibH1 and RibH2 share the same enzymatic activity since the *ribH1* gene could be exchanged with *ribH2*. RibH1 also could be replaced by the heterologous and characterized LS from *Saccharomyces cerevisiae* (LS_sce) [14] confirming that RibH1 and RibH2 are both active LSs *in vivo* (Figure 3Bi). As expected, p*ribH1*Km cannot be cured in the presence of an empty plasmid or with p*ribH2*_W22A (Figure 3Bii). RibH2_W22A contains a single amino acid mutation located in the active site of the endogenous RibH2. This mutant lacks enzymatic activity but its stability and structure are unaltered (unpublished results). Thus, these results confirm that *Brucella* needs at least one active LS to survive in riboflavin poor media. In agreement with this conclusion, the metabolic pressure to keep RibH1 can be overcome when very high levels of riboflavin (500 µM) are added to the media. Under these conditions, both the empty plasmid and the *ribH2*_W22A construct were able to replace RibH1 in the *ribH1-ribH2* mutant strain (Figure 3Biii). The same clones did not survive when plated in TSB-Amp media without the addition of riboflavin (Figure S2). Overall, these experiments demonstrate that the two *ribH* genes of *B. abortus* code for LS isoenzymes.

### RibH2 Activity Is Essential for *B. abortus* Intracellular Replication

Once established that both RibH1 and RibH2 can function as active LS *in vivo*, we decided to evaluate whether they play a role in virulence. For this purpose, we analyzed the intracellular replication of single and double RibH mutants in a macrophagic cell line and the persistence of these mutants in mice.

**Figure 4:**
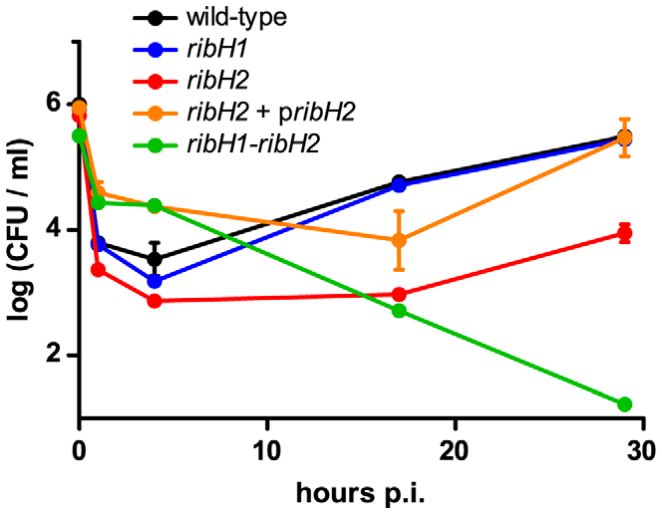
RibH2 is involved in survival and replication in J774A.1 macrophages. Macrophages infected with wild-type *B. abortus*, *ribH1*, *ribH2*, *ribH1-ribH2* mutants and the *ribH2* complemented strain (*ribH2*+p*ribH2*) were lysed and intracellular CFUs per ml quantified at different times after inoculation. Data shown (mean ± standard error of the mean) are representative of three independent experiments performed.

In J774 cells the wild-type strain showed a classical infection profile, with a marked decrease in viability between 0 and 4 h post-infection (p.i.) (Figure 4). This reflects cell entry and death by oxidative burst and fusion of the BCVs to lysosomes followed by a recovery phase where replication takes place after approximately 12 h p.i. (Figure 4). The *ribH1* strain showed a similar behavior to that of the wild-type strain. In contrast, *ribH2* showed a marked decrease in CFU counts at all time-points, suggesting that RibH2 is an important factor for intracellular life style. Complementation of *ribH2* by ectopic expression of RibH2 in a plasmid recovers this attenuated phenotype: this strain exhibited an increased survival at early times (1 and 4 h p.i.) and a similar curve to that of the wild-type strain at later times. These results are indicative that RibH2 is more important in the intracellular phase than RibH1. In spite of the fact that the double *ribH* mutant is unable to synthesize flavins, its intracellular flavin pool content is enriched when it is grown in the presence of 500 µM riboflavin (as analyzed by thin layer chromatography, results not shown). In clear agreement, the double *ribH* mutant exhibited an increased survival at early time points, but is unable to replicate at later times p.i. (Figure 4). This is indicative that flavin biosynthesis is essential for metabolic adaptation to the replicative niche.

**Figure 5:**
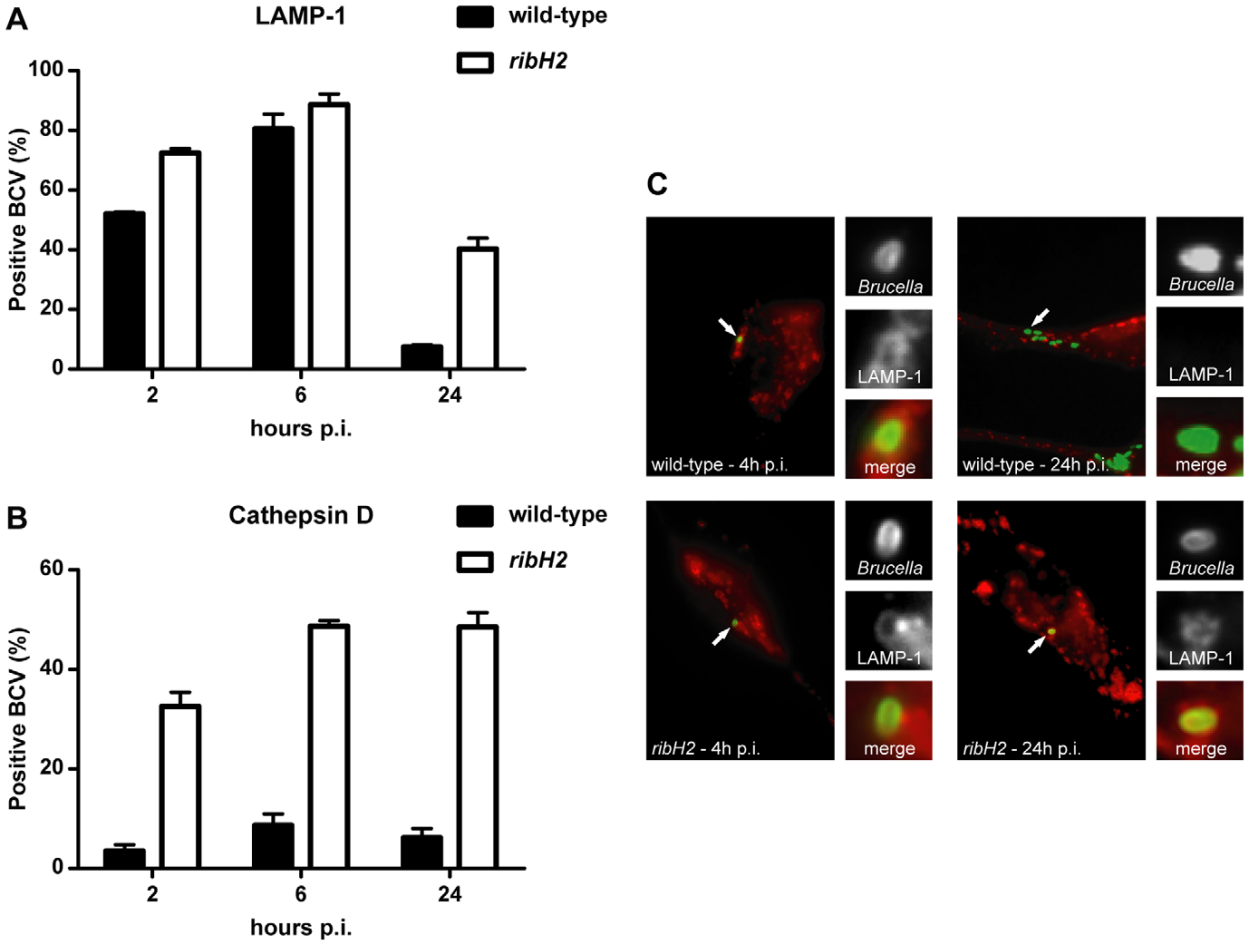
RibH2 is required to control BCV maturation in HeLa cells. HeLa cells were infected with *B. abortus* wild-type (black bars) or *ribH2* (white bars) strains expressing GFP and were labeled with (**A**) anti-cathepsin D or (**B**) anti-LAMP-1 antibodies at 2, 6 and 24 h p.i. (**C**) Representative fluorescence microscopy images at 4 and 24 h p.i. labeled with anti-LAMP-1 antibodies. White arrows show BCVs in infected HeLa cells. For (**A**) and (**B**) each determination is the average of two independent experiments. Values are expressed as mean ± standard error of the mean.

To further characterize the mechanisms by which RibH2 acts in intracellular survival, we analyzed the biogenesis of *ribH2* BCVs in HeLa cells by scoring the recruitment kinetics of the late endosome/lysosome glycoprotein LAMP-1 and the lysosomal luminal hydrolase cathepsin D. As previously described, LAMP-1 is rapidly acquired and then gradually excluded from wild-type BCVs [3]. Accordingly, at 24 h p.i. the wild-type BCVs were able to promote the maturation of replicative organelles and proliferated in LAMP-1 negative compartments. In contrast, the *ribH2* BCVs acquired this glycoprotein with similar kinetics but 40,3%±5,0 of BCVs retained LAMP-1 at 24 h, suggesting that this mutant fails to control vacuole maturation in HeLa cells (Figure 5A, 5C). As for cathepsin D, the percentage of *ribH2* positive BCVs vacuoles increased from 2 to 6 h p.i.. Additionally, at 24 h p.i. almost 50% of the phagosomes were positive for cathepsin D, whereas most of the wild-type BCVs excluded this molecule at the time points analyzed (Figure 5B). These results are consistent with bacterial survival at 4 and 48 h p.i. in HeLa cells as assessed by CFU counting (Figure S3). However, the number of *ribH2* CFUs retrieved at 48 h p.i. indicates that although the *ribH2* mutant is less efficient to promote the BCV maturation in comparison to the wild-type strain, it is capable of further survival and replication.

**Figure 6:**
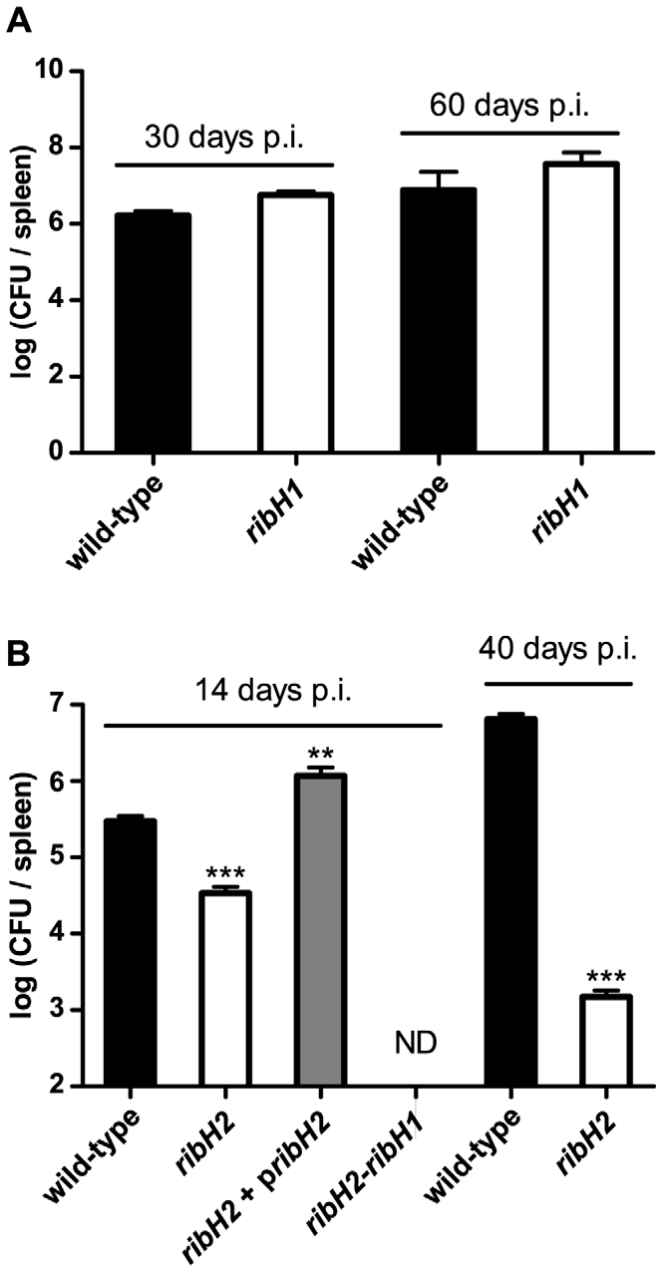
RibH2 mutant is attenuated in BALB/c mice infection. (**A**) BALB/c mice were infected with wild-type *B. abortus* (black bars), *ribH1* mutant (white bars) or (**B**) *ribH2* mutant (white bars), complemented *ribH2* strain (grey bars) or double *ribH* mutant (ND). After 14, 30, 40 or 60 days p.i. animals were sacrificed and spleens removed and processed for CFU counts as described in Materials and Methods. Values are expressed as mean ± standard deviation of the mean (N = 4). Student's unpaired two-tailed *T*-test was performed testing against wild-type strain in all cases. ND: not detected, asterisks indicate differences from wild-type infection treatment at the corresponding time: ** *p*<0.01, *** *p*<0.001.

The *ribH* mutants were also tested in mice infection. In agreement with cell experiments, *ribH1* behaved exactly as the wild-type strain (Figure 6A) showing the expected peak in CFU per spleen count at 14 days p.i. corresponding to the acute phase. Although we have not tested the effect at longer times, even at 60 days p.i. *ribH1* had no differences compared to the wild-type strain. In contrast, the *ribH2* strain showed a significant attenuation at all times tested with a spleen bacterial count 100-fold lower compared to the wild-type at 10 days p.i. and an even larger drop at 40 days p.i. (1000-fold, Figure 6B). The complemented *ribH2*+ p*ribH2* strain evidenced a larger bacterial count than the wild-type strain at 14 days p.i, demonstrating that RibH2 is required for survival in mice. The double mutant lacking both *ribH* genes was completely cleared by infected mice by day 14 p.i., indicating again that the biosynthesis of flavins is essential for survival (Figure 6B).

## Discussion

It is very difficult to characterize the main activities that support *Brucella* virulence by means of classical biochemical or microbiological studies. The main reason for this difficulty is the fact that *Brucella* behaves very differently in culture than inside cells or animal hosts. For these reasons, several genomic or proteomic approaches have been used to detect the genes that are important for virulence and pathogenesis. In some of these studies, *ribH2* has been identified as a differentially expressed gene. Al Dahouk *et al.* have detected that RibH2, among other proteins, is present in higher concentrations in intramacrophagic *Brucellae* than in culture [15]. Also, Lamontagne *et al.* made a comparative protein expression analysis between the attenuated S19 and the infectious 2308 *Brucella* strains where RibH2 was again identified to be augmented in the virulent strain [16]. In fact, RibH2 was originally characterized as a serological marker of active infection of both human and bovine brucellosis, implying that this protein is highly expressed during the infectious process [17]. Remarkably, the LS function is encoded by two different genes in *Brucella*, with *ribH1* located within the rib operon in chromosome I, and *ribH2* located in chromosome II (Table 1). Chromosome I is thought to code mainly housekeeping functions and chromosome II is regarded to harbor virulence-related functions that are differentially expressed during adaptation to the replicative niche inside cells [18]. Also, RibH2 has a very unusual regulatory element, the RFN element, which is supposedly a riboswitch found by bioinformatic methods [19] that down-regulates the translation of the protein by sensing the FMN concentration [20], [21]. Thus, the presence of this regulatory element added to the experimental evidence shown in this and other works are indicative that RibH2 harbors an important role in *Brucella* virulence.

**Table 1:** Comparison between RibH1 and RibH2 from *B. abortus*.

	RibH1	RibH2	Reference
Monomer Lenght (residues)	157	158	[22], [34]
Classification	Type-I LS	Type-II LS	[11], [22]
Quaternary arrangement	Pentamer	Decamer	[11], [22], [35]
LS *in vitro* k cat (s-1)	0.005 s-1 (very low)	0.006 s-1 (very low)	[11]
Chromosome location	Chromosome I	Chromosome II	[11]
Part of the Rib operon	Yes	No	[11]
Predicted regulatory element	-	RFN	[11]
Involved in host immune response	No	Yes	[17]
Involved in virulence	No	Yes	This work

**Figure 7:**
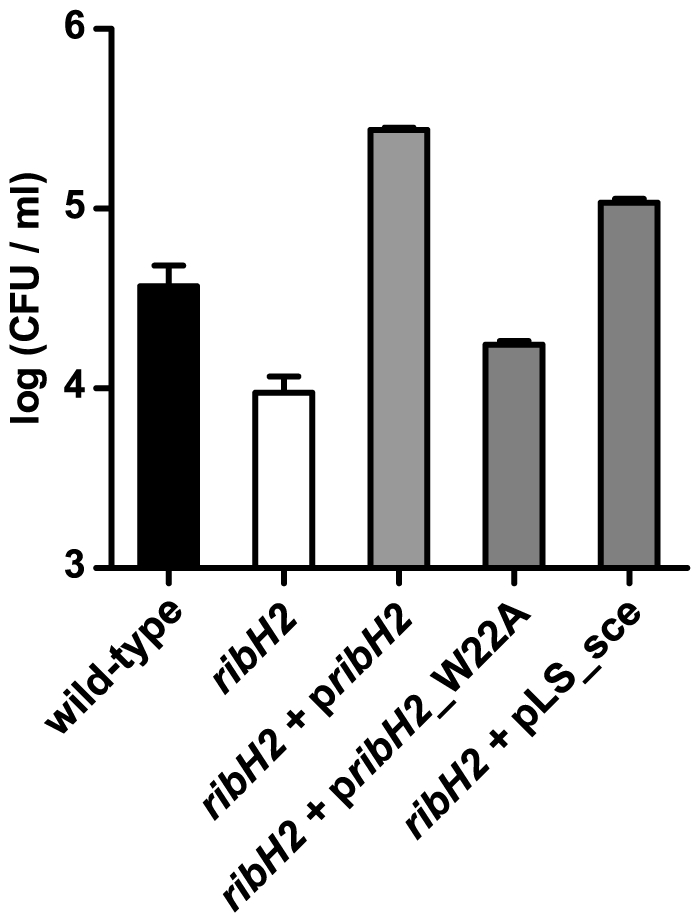
LS enzymatic activity of RibH2 is essential for *Brucella* survival. Wild-type *B. abortus* (black bar), *ribH2* mutant (white bar) and complemented *ribH2* strains (grey bars) with pBBR4 plasmid harboring different inserts (*ribH1*, *ribH2*, LS_sce, *ribH2*_W22A) were tested for survival at 4 h p.i. in J774A.1 macrophages. Data shown (mean ± standard error of the mean) are representative of three independent experiments performed.

Although *Brucella* RibH1 and RibH2 share only 21% of protein sequence identity, their monomers are structurally very similar, with a very low root mean square deviation (R.M.S.D.) of 1.50 Å when 128 C^α^ are aligned from a total of 157–158 C^α^. We have previously speculated that one plausible explanation for the important role of RibH2 in *Brucella* virulence is that this new type of decameric LS could harbor a novel yet unknown function [22]. To address this hypothesis we complemented the *ribH2* attenuated strain with different constructs (Figure 7). When *ribH2* is complemented with an active LS (endogenous RibH2 or the heterologous LS from yeast), the bacteria not only recover their virulence but also acquire an increased capacity to survive inside macrophages. In clear contrast, the complementation with the point-mutated RibH2_W22A does not recover the attenuated phenotype. Thus, it is unlikely that RibH2 is exerting a function different than LS.

Although the expression of two very similar LSs with the same function in *Brucella* is an intriguing observation, our working hypothesis is that the two *ribH* genes may be differentially expressed during the *Brucella* infection cycle. *Brucella* would use RibH1 for flavin biosynthesis during the extracellular phase and RibH2 during intracellular growth. In any case, the role of RibH2 points out to the importance of the biosynthetic pathway of flavins in virulence. We speculate that the LS activity could be a key point for the regulation of this pathway. In order to corroborate this hypothesis we are currently studying the regulation of *ribH* genes under different conditions.

As clearly shown in Figure 3, the *ribH1-ribH2* mutant is only viable at very high concentrations of exogenous flavins. This result supports previous bioinformatic analyses that indicate that *Brucella* lacks riboflavin transport activities. We infected cells with the *ribH1-ribH2* strain previously grown at high levels of riboflavin (Figure 4). Strikingly, these bacteria show an increased capacity to survive inside macrophages within the first stages of infection (supposedly because of their elevated intracellular flavin pool) but then they show an exponential drop in intracellular CFU counts until they are completely eliminated from the cells. This result may indicate that during the infection there is a high demand for flavins and without endogenous biosynthesis the internal pool is depleted within a few hours. Flavins would be essential for *Brucella* lifestyle inside the replicative niche where bacteria are isolated and need to survive to starvation and microaerobic respiration. Because there are no LS enzymes present in animals, *Brucella* LSs constitute very interesting targets for chemotherapy. LS inhibitors could lead to bacterial riboflavin auxotrophy or to attenuated bacterial virulence without endangering the mammalian host.

Two recent discoveries involving flavins in virulence and symbiosis in Rhizobiales have raised particular interest: Taga *et al.* demonstrated that in the symbiont *Sinorhizobium meliloti* the last step of biosynthesis of vitamin B12 is based on the fragmentation of FMN through the flavin destructase BluB, and Swartz *et al.* demonstrated that *B. abortus* contains a light-activated histidine kinase that binds a flavin chromophore and undergoes photochemistry indicative of cysteinyl-flavin adduct formation. This latter protein (BA-LOV-HK) appears to function as a photoreceptor that is directly related to *Brucella* survival and replication within macrophages [23], [24]. Thus, the role of flavin metabolism in *Brucella* pathogenesis is a question of biological relevance and appears not to be limited to the genus *Brucella*. We have performed bioinformatic and biochemical analyses in other bacteria [11], [22]. Many plant symbionts like *Rhizobium*, *Sinorhizobium*, *Mesorhizobium and Bradyrhizobium spp.* and some pathogens like *Ochrobactrum anthropi*, *Pseudomonas syringae pv. tomato* and *Roseobacter denitrificans* also present two *ribH* genes in their genomes exhibiting one Type-I and one Type-II LS. They are located in the respective genomes in the same fashion as in *Brucella*, meaning that *ribH1* is coded within the rib operon while *ribH2* is located as an isolated ORF with the regulatory RFN element upstream. A Type-I LS-*ribH* gene duplication might have occurred in a bacterial ancestor where one of these genes evolved to a Type-II LS, providing positive fitness for symbiosis or pathogenesis. Vertical and horizontal gene transfer could be responsible for the distribution of the *ribH* genes through the different taxa where two different types of LS are found.

Two mammal pathogens, *Rhodococcus equi* and *Actinobacillus pleuropneumoniae*, become avirulent when their riboflavin operons are disrupted [25], [26], and therefore these mutants seem to be good candidates for live attenuated vaccines [27], [28]. For these reasons the *ribH1-ribH2* strain generated in this work could also be a very good candidate for developing a *Brucella* vaccine because it can not synthesize riboflavin and it is completely attenuated in cells and in mice. This strain also lacks the serological marker RibH2 which could eventually be used to discriminate between vaccinated and infected animals [29], [30]. In summary, in this work we show that flavin biosynthesis is essential for *Brucella* survival inside cells and that the LSs constitute attractive targets for chemotherapy and vaccine design.

## Materials and Methods

### Bacterial Strains

All live *Brucella* strains have been manipulated in a BSL-3 facility at UNSAM University. Bacterial strains, plasmids and oligonucleotides used in this study are listed in Tables 2, 3 and 4. *B. abortus* strains were grown in Tryptic Soy Broth (TSB, BD), Tryptic Soy Agar (TSA, BD) or Gerhardt-Wilson minimal medium at 37°C on a rotary shaker (250 rpm) or stove for plates [31]. When necessary, the following antibiotics were added to the indicated final concentrations: kanamycin (Km), 50 µg/ml; gentamicin (Gm), 3 µg/ml); carbenicillin (Cb), 50 µg/ml, nalidixic acid (Nal), 5 µg/ml; and ampicillin (Amp), 50 µg/ml. *Escherichia coli* strains were grown at 37°C on a rotary shaker (250 rpm) in Luria-Bertani broth. For *E. coli* strains antibiotics were used at the following concentrations: Km, 50 µg/ml; Gm, 20 µg/ml; Amp, 100 µg/ml and streptomycin (Str), 25 µg/ml.

**Table 2:** *B. abortus* and *E .coli* strains used in this study.

Strain	Name	Characteristics	Source or reference
*B. abortus* strains	2308	wild-type, smooth, virulent, Nal^r^	Laboratory stock
	2308 pGFP	2308, containing plasmid pGFP, Km^r^	This work
	*ribH1*	2308, unmarked deletion of *ribH1*	This work
	*ribH1*+p*ribH1*	*ribH1*, containing plasmid p*ribH1*, Amp^r^	This work
	*ribH2*	2308, Gm^r^, insertion of acc(3)-I cassette in *ribH2*	This work
	*ribH2* pGFP	*ribH2*, containing plasmid pGFP, Km^r^	This work
	*ribH2*+p*ribH2*	*ribH2*, containing plasmid p*ribH2*, Amp^r^	This work
	*ribH1-ribH2*+p*ribH1*Km	*ribH1*, *ribH2*, containing plasmid p*ribH1*Km, Km^r^	This work
	*ribH1-ribH2*	*ribH1*, *ribH2*	This work
*E. coli* strains	S17-1	*recA pro hsdR* RP4-2-Tc::Mu-Km::Tn7	[33]

**Table 3:** Plasmids used in this study.

Name	Characteristics	Source of reference
pBBR4	pBBR1MCS-4 Broad-host-range cloning vector, Amp^r^	[32]
pBBR2	pBBR1MCS-2 Broad-host-range cloning vector, Km^r^	[32]
pGemT*ribH2*	0.9 Kbp EcoRI fragment containing *ribH2* gene, cloned into pGemT-Easy vector, Amp^r^	This work
p*ribH1*Amp	1 Kbp EcoRI fragment containing *ribH1*, cloned into pBBR1MCS-4	This work
p*ribH1*Km	1 Kbp EcoRI fragment containing *ribH1*, cloned into pBBR1MCS-2	This work
p*ribH2*	0.9 Kbp EcoRI fragment containing *ribH2* gene, cloned into pBBR1MCS-4	This work
p*ribH2*_W22A	point mutant W22A ribH2 gene, cloned into pBBR1MCS-4	This work
pLS_sce	RIB4 gene from *Sacharomyces cerevisiae*, cloned into pBBR1MCS-4	This work
pGFP	gfp-mut3 cloned into pBBR1MCS-2	This work
pBluescript-KSII	Commercial plasmid	Stratagene
pGem-T-Easy	Commercial plasmid	Promega
pBlueKSIIsacBΔ*ribH1*	Δ*ribH1* deletion allele and sacB from *B. subtilis*, cloned into pBluescript-KSII	This work
pGem-T*ribH2*::acc(3)-1	pGem-T*ribH2*::acc(3)-I	This work

**Table 4:** Oligonucleotides used in this study.

Name	Sequence^a^	Restriction Enzyme
ribH1_F1	CGGGATCCGCGCCTTTCATCGCGCAGAA	BamHI
ribH1_R1	TCCAGACTGCTACGTATCGCCTTGGACATGAGAAACTCCAT	-
ribH1_F2	GCGATACGTAGCAGTCTGGACGCAAAAAATTCGGAGCCTGA	-
ribH1_R2	GGACTAGTCGCGACAGCGGCCAGTCTTC	SpeI
ribH2_F1	GGAGAATGCGTGATAAATTCAA	-
ribH2_R1	TCAGACAAGCGCGGCGAT	-
ribH2_F2	TTAAGGATCCATGGCTAGCAACCAAAGC	BamHI
ribH2_R2	ATTAGGATCCGCTAGCTCAGACAAGCG	BamHI
LS_sce_F	ATTACTCGAGATGCATCACCATCACCATCACGCAGTTAAAGGATTAGGCAAAC	XhoI
LS_sce_R	CGATTCTAGATCAAAAAGCATTTTTACCGAAC	XbaI
W22A_F	ATTCAGGCCCGCGCCCACGCCGACATC	-

a Restriction endonuclease cleavage sites are underlined.

### Construction of the *B. abortus* Mutants

#### *ribH1* mutant

For the construction of the ORF BAB1_0791 (*ribH1*) unmarked chromosomal mutant two PCR fragments were generated from regions flanking this ORF. Oligonucleotides *ribH1*_F1 and *ribH1*_R1 were used to amplify a 0.3 Kbp fragment including codons 1–7 and oligonucleotides *ribH1*_F2 and *ribH1*_R2 were used to amplify a 0.3 Kbp fragment including 150–157 codons of *ribH1*. Both fragments (containing complementary regions) were ligated by overlapping PCR using oligonucleotides *ribH1*_F1 and *ribH1*_R2. The resulting fragment containing the *ribH1* deletion allele was cloned into pBluescript-KSII (Stratagene) in which the *Bacillus subtilis* sacB gene encoding levansucrase (that induces lethality upon exposure to 5% (w/v) sucrose in the growth medium) had been cloned in the PstI site to generate pBlueKSIIsacBΔ*ribH1*. This plasmid was introduced in *B. abortus* 2308 by electroporation. The first recombination event (integration of the suicide vector in the chromosome) was selected by Cb resistance and 10% (w/v) sucrose sensitivity in TSA plates. Cells were grown overnight in TSB in the absence of antibiotic selection to promote recombination. The second recombination event (excision of the plasmid and generation of the mutant strain by allelic exchange) was selected by Cb sensitivity and 10% (w/v) sucrose resistance in TSA plates. Colonies were screened by PCR using oligonucleotides *ribH1*_F1 and *ribH1*_R2 that amplify a fragment of 0.6 Kbp in the mutant strain and a fragment of 1.2 Kbp in the wild-type strain. The mutant strain carrying an unmarked deletion of *ribH1* was called *ribH1*.

#### *ribH2* mutant

A PCR product of 0.9 Kbp containing ORF BAB2_0545 (*ribH2*) was amplified using oligonucleotides *ribH2*_F1 and *ribH2*_R1 and ligated to pGem-T-Easy vector (Promega) to generate pGem-T*ribH2*. The plasmid was linearized with EcoRV. Linearized pGem-T*ribH2* was ligated to a 0.7 Kbp SmaI fragment containing a acc(3)-I cassette to generate pGem-T*ribH2*::acc(3)-I. This plasmid was electroporated into *B. abortus* 2308 where it is incapable of autonomous replication. Homologous recombination events were selected using Gm resistance (3 µg/ml) and Cb sensitivity (50 µg/ml) in TSA plates. PCR and sequencing analyses showed that the *ribH2* wild-type gene was replaced by the disrupted one. The mutant strain obtained was called *ribH2*.

#### *ribH1*-*ribH2* mutant

A 1 Kbp PCR product containing *ribH1* gene was first cloned into pGem-T-Easy and then excised and ligated to the EcoRI site of pBBR2 [32] to generate p*ribH1*Km. This plasmid was conjugated into the *ribH1* strain by biparental mating to generate *ribH1*+p*ribH1*Km. Then pGem-T*ribH2*::acc(3)-I plasmid was electroporated into this strain and homologous recombination events were selected as previously described. PCR and sequencing analyses showed that the *ribH2* wild-type gene was replaced by the disrupted one. The mutant strain obtained was called *ribH1-ribH2*+p*ribH1*Km.

#### Conjugation by biparental mating

*E. coli* S17-1 [33] donor strain and *B. abortus* strains were cultured separately, cells were harvested during the exponential phase and 1 ml of each culture was pelleted and washed twice with TSB. Bacteria were then centrifuged, resuspended in residual liquid, mixed and plated together in a single spot in a TSA plate without antibiotics. Plates were incubated at 37°C overnight, afterwards the mix was plated in TSA-Nal plates to select *Brucella* and exconjugants were selected for the appropriate plasmidic antibiotic resistance.

### Construction of the Complemented *ribH2* Strains

#### RibH1

A 1 Kbp PCR product containing the *ribH1* gene was first cloned into pGem-T-Easy and then excised and ligated to the EcoRI site of pBBR4 [32] to generate p*ribH1* where *ribH1* is under the lac promoter. This plasmid was conjugated into the *ribH2* strain by biparental mating to generate *ribH1 *+ p*ribH1*Amp.

#### RibH2

A 0.9 Kbp EcoRI fragment containing *ribH2* gene was excised from pGem-T*ribH2* and ligated to the EcoRI site of pBBR4. The resulting plasmid, p*ribH2*, was conjugated into the *ribH2* strain by biparental mating to generate *ribH2 *+ p*ribH2*.

#### LS_sce

ORF YOL143C was amplified using the oligonucleotides LS_sce_F and LS_sce_R from *S. cerevisiae* genomic DNA. The resulting 0.6 Kbp PCR fragment was purified and digested with XhoI and XbaI and ligated into pBBR4 digested with the same restriction enzymes. The resulting plasmid, pLS_sce, was confirmed by sequencing and conjugated into *ribH2* strain by biparental mating to generate *ribH2 *+ pLS_sce.

#### RibH2_W22A

The *ribH2*_W22A mutation was generated using the oligonucleotides W22A_F and *ribH2*_R2 to amplify the first part of the *ribH2* ORF, then the PCR product was purified and used as a “megaprimer” for a second amplification round using the oligonucleotide *ribH2*_F2. The resultant PCR product was purified, digested with BamHI and ligated to pBBR4 digested with the same restriction enzyme. The resulting plasmid, p*ribH2*_W22A, was confirmed by sequencing and conjugated into *ribH2* strain by biparental mating to generate *ribH2 *+ pLS_sce.

#### Plasmid swap

To demonstrate that RibH1 and RibH2 are LSs *in vivo*, we developed a plasmid swap experiment based the instability of two vectors from the same incompatibility group harbored in the same bacterial population. To this end, a pBBR4 plasmid harboring different inserts (*ribH1*, *ribH2*, *ribH2*_W22A, LS_sce or an empty plasmid) was introduced by biparental mating conjugation into the *ribH1-ribH2 *+ p*ribH1*Km strain. We selected the presence of both vectors with Amp and Km. Three independent Amp^r^/Km^r^ clones from each conjugation were cultured for at least 15 generations in TSB added with Amp only, then dilutions were plated in TSA-Amp plates. One hundred colonies from these plates were replica plated in TSA with Amp or Km. When necessary, riboflavin (500 µM) was added to the TSB or TSA. The percentage of Amp^r^/Km^s^ clones was calculated for each case (n = 100).

#### Bacterial infection assays

Monolayers of J774.A1 cells were seeded in 24-well plates (10^5^ cells per well) and inoculated with RPMI 1640 supplemented with 5% fetal calf serum (FCS) containing 5×10^5^ bacteria (MOI = 5∶1). In order to ensure close contact between cells and bacteria, multiwell plates were centrifuged at 400 g for 10 min After 30 min of incubation at 37°C in a 5% CO_2_ atmosphere, cells were gently washed with phosphate buffered saline (PBS, pH 7.4) and then incubated for 1 h in medium supplemented with Str (100 µg/ml) and Gm (50 µg/ml) to kill remaining extracellular bacteria. Thereafter, the antibiotic concentration was decreased to 20 µg/ml of Str and 10 µg/ml of Gm. At the indicated times p.i., the number of intracellular viable bacteria was determined as follows: cells were washed three times with PBS and treated for 5 min with 0.5 ml of 0.1% Triton X-100. Lysates were serially diluted and plated on TSA plates with the appropriate antibiotic to determine colony forming units per ml (CFU/ml). Each determination is the average of two independent experiments. Values are expressed as mean ± standard error of the mean.

HeLa cells were seeded in coverslips (10^5^ cells per coverslip) and inoculated with minimal essential medium (MEM; Gibco) supplemented with 5% FCS and 2 mM glutamine containing 5×10^7^ CFU of bacteria (MOI = 500∶1). Multiwell plates were centrifuged at 400 g for 10 min After 1 h of incubation at 37°C in a 5% CO_2_ atmosphere, cells were gently washed with PBS (pH 7.4) and then incubated for 1 h in medium supplemented with 100 µg/ml of Str and 50 µg/ml of Gm to kill remaining extracellular bacteria. Thereafter, the antibiotic concentration was decreased to 20 µg/ml of Str and 10 µg/ml of Gm. At the indicated times p.i. cells were washed and fixed for immunofluorescence.

#### Immunofluorescence microscopy

HeLa cells were washed five times with PBS to remove non-adherent bacteria and fixed for 15 min in 3% paraformaldehyde (pH 7.4) at 37°C and then processed for immunofluorescence labeling. Briefly, coverslips were washed three times with PBS, incubated for 10 min with PBS added with 250 mM NH_4_Cl in order to quench free aldehyde groups. Coverslips were then incubated with primary antibodies in a PBS 10% horse serum 0.1% saponin solution for 20 min at room temperature, washed in PBS containing 0.1% saponin and then incubated with secondary antibodies in a PBS 10% horse serum 0.1% saponin solution. The coverslips were mounted onto glass slides using Mowiol (Aldrich). Cells were observed on the microscope using a 100× oil immersion objective. Projections were saved in TIFF format and imported to ADOBE PHOTOSHOP CS where images were merged using RGB format. To determine the percentages of bacteria that co-localized with the studied intracellular markers a minimum of 100 intracellular bacteria (revealed by indirect immunofluorescence) were counted. The assays were performed in duplicate.

#### Antibodies and reagents

For immunofluorescence, the primary antibodies used were mouse anti-human LAMP-1 H4A3 (Developmental Studies Hybridoma Bank, Department of Biological Sciences, University of Iowa) or rabbit anti-human cathepsin D (Dako). The secondary antibodies used were goat anti-mouse or goat anti-rabbit Alexa Fluor 568 (Molecular Probes, Invitrogen Co.). For DNA staining, Hoechst dye at 2 µg/ml (final concentration) was used. For Western Blots, the primary antibodies used were monoclonal anti-RibH2 and polyclonal anti-RibH1 mice serum (both generated in our laboratory). The secondary antibody used was goat anti-mouse IgG (Fc Specific) Peroxidase Conjugate (Sigma).

#### Western blots

Bacteria were grown overnight with the proper antibiotics and extra added riboflavin when necessary. The same amount of protein was loaded into each lane in the gels. Chemiluminiscent ECL Plus Western Blotting Detection System (Amersham Biosciences), Storm Image and Detection system (Molecular Dynamics) and Immobilon-NC Transfer Membranes (Millipore) were used for the process.

#### Mice infection assays

60-day-old female BALB/c mice were inoculated intraperitoneally with 5×10^5^ CFU of the indicated strains. At 14, 30, 40 or 60 days p.i. mice were sacrificed and spleens were removed and homogenized in 2 ml of PBS. Tissue homogenates were serially diluted and plated on TSA plates with the appropriate antibiotics to determine colony forming units per spleen (CFU/spleen). Values are expressed as mean ± standard errors of the mean (N = 4).

All research involving animals has been conducted according to the National Institutes of Health Guide for the Care and Use of Laboratory Animals, and all experimental protocols have been approved by the Institutional Animal Care and Use Committee (IACUC) of Leloir Institute.

#### Statistical analysis

Data are presented as mean ± standard error of the mean. Significance was reported using Student's unpaired two-tailed *T*-test (Prism, GraphPad Software). *p*-values<0.05 were considered statistically relevant.

## Supporting Information

Figure S1 Plants, fungi, and microorganisms share a common flavin biosynthetic pathway. Animals take riboflavin as a vitamin in their diets to synthesize FMN and FAD cofactors (by RK and FS respectively).
(2.12 MB EPS)

Figure S2 Amp^r^/Km^s^ clones carrying empty pBBR4 or p*ribH2*_W22A derived from the plasmid swap experiment were plated in TSB-Amp plates without the addition of riboflavin. Control replica plates in TSB-Km plates with riboflavin added are also shown.
(1.32 MB TIF)

Figure S3 HeLa cells infected with wild-type *B. abortus* (black) and *ribH2* mutant (red) were lysed and intracellular CFUs per ml quantified at different times after inoculation. Data shown (mean ± standard error of the mean) are representative of three independent experiments performed.
(2.02 MB TIF)

## References

[1] Pappas G, Papadimitriou P, Akritidis N, Christou L, Tsianos EV (2006). The new global map of human brucellosis.. Lancet Infect Dis.

[2] Roop RM, Bellaire BH, Valderas MW, Cardelli JA (2004). Adaptation of the Brucellae to their intracellular niche.. Mol Microbiol.

[3] Pizarro-Cerda J, Meresse S, Parton RG, van der Goot G, Sola-Landa A (1998). Brucella abortus transits through the autophagic pathway and replicates in the endoplasmic reticulum of nonprofessional phagocytes.. Infect Immun.

[4] Celli J, de Chastellier C, Franchini DM, Pizarro-Cerda J, Moreno E (2003). Brucella evades macrophage killing via VirB-dependent sustained interactions with the endoplasmic reticulum.. J Exp Med.

[5] Starr T, Ng TW, Wehrly TD, Knodler LA, Celli J (2008). Brucella intracellular replication requires trafficking through the late endosomal/lysosomal compartment.. Traffic.

[6] Celli J (2006). Surviving inside a macrophage: the many ways of Brucella.. Res Microbiol.

[7] Joosten V, van Berkel WJ (2007). Flavoenzymes.. Curr Opin Chem Biol.

[8] Jimenez de Bagues MP, Loisel-Meyer S, Liautard JP, Jubier-Maurin V (2007). Different roles of the two high-oxygen-affinity terminal oxidases of Brucella suis: Cytochrome c oxidase, but not ubiquinol oxidase, is required for persistence in mice.. Infect Immun.

[9] Fischer M, Bacher A (2005). Biosynthesis of flavocoenzymes.. Nat Prod Rep.

[10] Fischer M, Bacher A (2008). Biosynthesis of vitamin B2: Structure and mechanism of riboflavin synthase.. Arch Biochem Biophys.

[11] Zylberman V, Klinke S, Haase I, Bacher A, Fischer M (2006). Evolution of vitamin B2 biosynthesis: 6,7-dimethyl-8-ribityllumazine synthases of Brucella.. J Bacteriol.

[12] Imlay JA (2002). How oxygen damages microbes: oxygen tolerance and obligate anaerobiosis.. Adv Microb Physiol.

[13] Klinke S, Zylberman V, Vega DR, Guimaraes BG, Braden BC (2005). Crystallographic studies on decameric Brucella spp. Lumazine synthase: a novel quaternary arrangement evolved for a new function?. J Mol Biol.

[14] Garcia-Ramirez JJ, Santos MA, Revuelta JL (1995). The Saccharomyces cerevisiae RIB4 gene codes for 6,7-dimethyl-8-ribityllumazine synthase involved in riboflavin biosynthesis. Molecular characterization of the gene and purification of the encoded protein.. J Biol Chem.

[15] Al Dahouk S, Jubier-Maurin V, Scholz HC, Tomaso H, Karges W (2008). Quantitative analysis of the intramacrophagic Brucella suis proteome reveals metabolic adaptation to late stage of cellular infection.. Proteomics.

[16] Lamontagne J, Forest A, Marazzo E, Denis F, Butler H (2009). Intracellular adaptation of Brucella abortus.. J Proteome Res.

[17] Goldbaum FA, Leoni J, Wallach JC, Fossati CA (1993). Characterization of an 18-kilodalton Brucella cytoplasmic protein which appears to be a serological marker of active infection of both human and bovine brucellosis.. J Clin Microbiol.

[18] Viadas C, Rodriguez MC, Garcia-Lobo JM, Sangari FJ, Lopez-Goni I (2009). Construction and evaluation of an ORFeome-based Brucella whole-genome DNA microarray.. Microb Pathog.

[19] Abreu-Goodger C, Merino E (2005). RibEx: a web server for locating riboswitches and other conserved bacterial regulatory elements.. Nucleic Acids Res.

[20] Gelfand MS, Mironov AA, Jomantas J, Kozlov YI, Perumov DA (1999). A conserved RNA structure element involved in the regulation of bacterial riboflavin synthesis genes.. Trends Genet.

[21] Vitreschak AG, Rodionov DA, Mironov AA, Gelfand MS (2004). Riboswitches: the oldest mechanism for the regulation of gene expression?. Trends Genet.

[22] Klinke S, Zylberman V, Bonomi HR, Haase I, Guimaraes BG (2007). Structural and kinetic properties of lumazine synthase isoenzymes in the order Rhizobiales.. J Mol Biol.

[23] Taga ME, Larsen NA, Howard-Jones AR, Walsh CT, Walker GC (2007). BluB cannibalizes flavin to form the lower ligand of vitamin B12.. Nature.

[24] Swartz TE, Tseng TS, Frederickson MA, Paris G, Comerci DJ (2007). Blue-light-activated histidine kinases: two-component sensors in bacteria.. Science.

[25] Fuller TE, Thacker BJ, Mulks MH (1996). A riboflavin auxotroph of Actinobacillus pleuropneumoniae is attenuated in swine.. Infect Immun.

[26] Ashour J, Hondalus MK (2003). Phenotypic mutants of the intracellular actinomycete Rhodococcus equi created by in vivo Himar1 transposon mutagenesis.. J Bacteriol.

[27] Fuller TE, Thacker BJ, Duran CO, Mulks MH (2000). A genetically-defined riboflavin auxotroph of Actinobacillus pleuropneumoniae as a live attenuated vaccine.. Vaccine.

[28] Lopez AM, Townsend HG, Allen AL, Hondalus MK (2008). Safety and immunogenicity of a live-attenuated auxotrophic candidate vaccine against the intracellular pathogen Rhodococcus equi.. Vaccine.

[29] Schurig GG, Sriranganathan N, Corbel MJ (2002). Brucellosis vaccines: past, present and future.. Vet Microbiol.

[30] Moriyon I, Grillo MJ, Monreal D, Gonzalez D, Marin C (2004). Rough vaccines in animal brucellosis: structural and genetic basis and present status.. Vet Res.

[31] Gerhardt P (1958). The nutrition of brucellae.. Bacteriol Rev.

[32] Kovach ME, Phillips RW, Elzer PH, Roop RM, Peterson KM (1994). pBBR1MCS: a broad-host-range cloning vector.. Biotechniques.

[33] Simon R, Priefer U, Pühler A (1983). A Broad Host Range Mobilization System for In Vivo Genetic Engineering: Transposon Mutagenesis in Gram Negative Bacteria.. Bio/Technology.

[34] Braden BC, Velikovsky CA, Cauerhff AA, Polikarpov I, Goldbaum FA (2000). Divergence in macromolecular assembly: X-ray crystallographic structure analysis of lumazine synthase from Brucella abortus.. J Mol Biol.

[35] Zylberman V, Craig PO, Klinke S, Braden BC, Cauerhff A (2004). High order quaternary arrangement confers increased structural stability to Brucella sp. lumazine synthase.. J Biol Chem.

