# Supplementary figures and images for: An Atypical Riboflavin Pathway Is Essential for Brucella abortus Virulence
Source: PLoS One. 2010 Feb 25;5(2):e9435. doi: 10.1371/journal.pone.0009435 (PMC2828483; doi:10.1371/journal.pone.0009435)

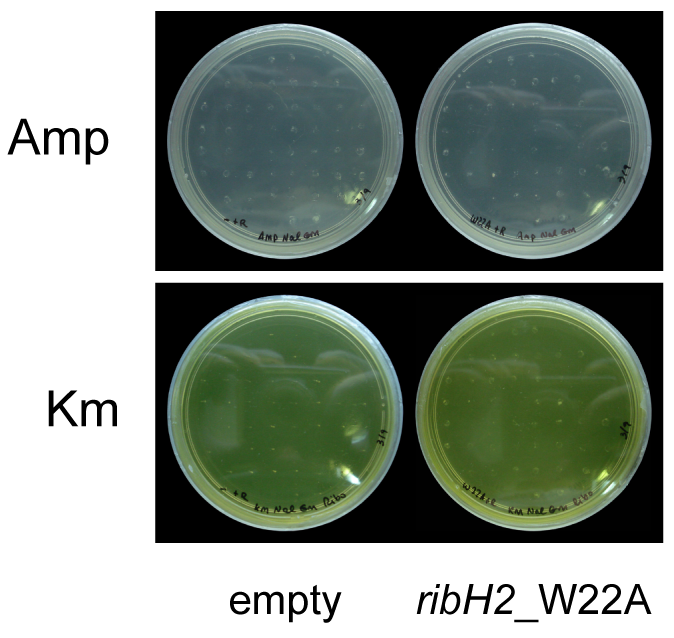

Supplement: Figure S2 — Ampr/Kms clones carrying empty pBBR4 or pribH2_W22A derived from the plasmid swap experiment were plated in TSB-Amp plates without the addition of riboflavin. Control replica plates in TSB-Km plates with riboflavin added are also shown. (1.32 MB TIF) [file pone.0009435.s002.tif]

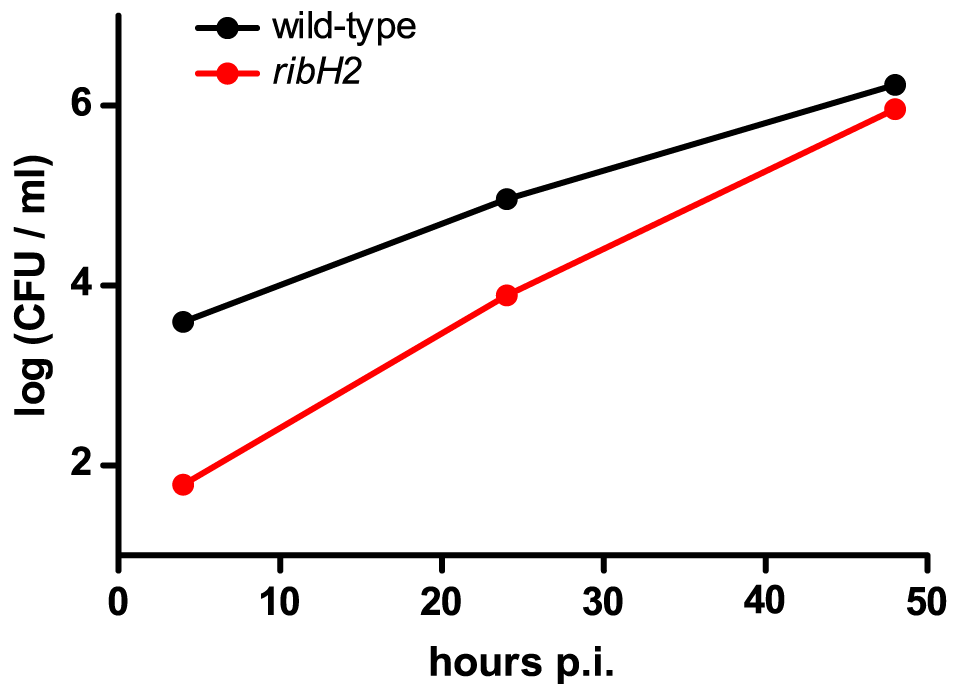

Supplement: Figure S3 — HeLa cells infected with wild-type B. abortus (black) and ribH2 mutant (red) were lysed and intracellular CFUs per ml quantified at different times after inoculation. Data shown (mean ± standard error of the mean) are representative of three independent experiments performed. (2.02 MB TIF) [file pone.0009435.s003.tif]
